# The real-world safety evaluation of the selective NaV1.8 inhibitor suzetrigine: disproportionality analysis and flexible empirical bayes signals from FAERS with external triangulation

**DOI:** 10.3389/fphar.2026.1814013

**Published:** 2026-06-11

**Authors:** Kun Lai, Lan Luo, Fuzhao Zhang, Eugenie Sin Sing Tan, Anand Gaurav, Jianghua Zheng, Chung Keat Tan

**Affiliations:** 1 Affiliated Hospital of North Sichuan Medical College, Nanchong, Sichuan, China; 2 Faculty of Medicine and Health Sciences, UCSI University Bandar Springhill Campus, Port Dickson, Negeri Sembilan, Malaysia; 3 Department of Pharmaceutical Sciences, School of Health Sciences and Technology, UPES, Dehradun, Uttarakhand, India; 4 Key Laboratory of General Surgery, Affiliated Hospital of North Sichuan Medical College, Nanchong, Sichuan, China

**Keywords:** disproportionality, empirical bayes, FAERS, NaV1.8 inhibitor, pharmacovigilance, post-marketing surveillance, sensory dysesthesia, suzetrigine

## Abstract

**Background:**

Suzetrigine (Journavx; VX-548), the first selective NaV1.8 voltage-gated sodium channel inhibitor approved by the FDA on 30 January 2025, represents a novel non-opioid option for moderate-to-severe acute pain. Given its recent market entry and unique peripheral mechanism, comprehensive post-marketing safety surveillance is essential.

**Objective:**

This study aimed to identify and characterize adverse event signals associated with suzetrigine in the FDA Adverse Event Reporting System (FAERS) using advanced disproportionality and comparator-referenced empirical Bayes (EB) methods, with external triangulation against published literature and WHO VigiBase data.

**Methods:**

We analyzed FAERS reports through the first 8 months post-approval. Disproportionality metrics (ROR, PRR, IC) were supplemented by a comparator-referenced EB profiling approach that incorporated suzetrigine, acetaminophen, ibuprofen, and background “other drugs,” generating 3,000 posterior draws per preferred term (PT). Signals with EB q05 > 2 and no comparator overlap were classified as suzetrigine-unique. High-priority PTs were triangulated with Phase II/III trial data, systematic reviews, case reports, and VigiBase report counts (February 2026).

**Results:**

Of 19 prioritized PTs, 14 were suzetrigine-unique. Dominant clusters included sensory disturbances (paresthesia, burning sensation, skin burning sensation, hypoaesthesia; EB q05 11.43–31.16), musculoskeletal events (muscle spasms EB q05 31.15), and cutaneous reactions (pruritus, rash). These signals were mechanistically consistent with peripheral NaV1.8 blockade of nociceptors and pruriceptors. Literature and VigiBase data corroborated neurological/sensory and musculoskeletal signals; psychiatric signals (euphoric mood, abnormal dreams) lacked external support and were deprioritized.

**Conclusion:**

This real-world pharmacovigilance analysis identifies a distinct safety signature for suzetrigine, with neurological and sensory disturbances (e.g., paresthesia, burning sensation, skin burning sensation, hypoaesthesia) emerging as prominent signals not fully characterized in pre-approval trials, whereas musculoskeletal and cutaneous events largely align with labeled reactions. These hypothesis-generating findings highlight the need for focused post-marketing surveillance on neurological/sensory preferred terms through prospective cohort studies and Phase IV trials to quantify incidence, identify risk factors, and optimize risk-minimization strategies for this promising non-opioid analgesic.

## 1 Introduction

Acute pain is among the most frequent clinical problems, yet achieving effective analgesia while reducing opioid-related harms remains difficult in everyday practice, sustaining demand for non-opioid options with novel mechanisms (Dowell et al., 2022). Suzetrigine (VX-548; marketed as JOURNAVX) is a first-in-class, peripherally acting NaV1.8 inhibitor intended to attenuate nociceptive signaling without activating central opioid pathways (Osteen et al., 2025). In established acute pain models, selective NaV1.8 inhibition produced clinically meaningful analgesia with adverse events that were generally mild-to-moderate in severity (Jones et al., 2023; Bertoch et al., 2025). Following U.S. regulatory approval for moderate-to-severe acute pain, defining suzetrigine’s real-world safety profile is a priority because pre-approval trials may be underpowered for rare events and may not capture heterogeneous postmarketing populations (U.S. Food and Drug Administration, 2025a; U.S. Food and Drug Administration, 2025b). Spontaneous reporting systems such as the FDA Adverse Event Reporting System (FAERS) can complement trials by enabling earlier detection of rare, unexpected, or population-specific adverse event patterns.

Nevertheless, signal detection in FAERS is intrinsically vulnerable to bias and data-quality constraints. FDA explicitly notes that duplicate and incomplete reports exist, that the presence of a report does not establish causality, and that reporting rates cannot be interpreted as incidence (FDA, 2025c). Duplicate reporting can be substantial and may affect both signal identification and evaluation (Janiczak et al., 2025). In addition, underreporting remains a pervasive limitation of spontaneous reporting, and systematic reviews highlight that both healthcare-professional and patient-level knowledge, attitudes, and practical barriers contribute to missed reports (Routledge and Bracchi, 2023; Costa et al., 2023). Temporal artifacts can further distort the reporting landscape (e.g., product lifecycle–related reporting changes), and external shocks such as the COVID-19 era can introduce masking or shifts in signal detectability within FAERS and other spontaneous-reporting systems (Arora et al., 2017; Montes-Grajales et al., 2023). Collectively, these issues motivate analytic designs that emphasize robustness and careful prioritization rather than single-pass “one-algorithm” screening.

To address these challenges, we constructed a dual-pathway signal detection strategy that balances statistical robustness with drug-specific interpretability. First, we performed PT-level screening using complementary disproportionality and empirical Bayes shrinkage approaches (e.g., ROR/PRR/IC alongside EBGM-type shrinkage), leveraging Bayesian shrinkage to stabilize estimates under sparse counts (Canida and Ihrie, 2017). Second, to better characterize drug-specificity within an analgesic context, we implemented a comparator-referenced empirical Bayes framework incorporating suzetrigine, acetaminophen, ibuprofen, and an “other drugs” background, comparing EB distributions for the same PT across exposures to identify patterns more consistent with a suzetrigine-driven signal than with background analgesic or indication-related reporting (Tan et al., 2025).

Accordingly, this study aimed to systematically characterize postmarketing adverse event signals associated with suzetrigine in FAERS by integrating (1) multi-testing–aware traditional disproportionality/Bayesian shrinkage screening with (2) comparator-referenced empirical Bayes profiling to prioritize candidate PTs for clinical interpretation. As a secondary aim, we sought to triangulate high-priority PTs using external evidence, including prior literature, publicly available FDA review/labeling and trial materials, and adverse event information disclosed in ClinicalTrials.gov, WHO vigibase, to enhance clinical credibility and translational relevance of detected signals (FDA, 2025a; FDA, 2025b; Bertoch et al., 2025).

## 2 Methods

### 2.1 Data source (FAERS)

We conducted a retrospective pharmacovigilance study using the U.S. Food and Drug Administration (FDA) Adverse Event Reporting System (FAERS). We downloaded the publicly available quarterly FAERS data files covering 2025Q1–2025Q3 from the FDA Quarterly Data Extract (QDE) portal (U.S. Food and Drug Administration, 2025d).

FAERS is a spontaneous reporting system designed for postmarketing safety surveillance; reports are voluntarily submitted and do not establish causality, and duplicate/updated case versions may occur (U.S. Food and Drug Administration, 2023).

To reduce duplication, we deduplicated reports at the case level using a commonly applied FDA-recommended approach: reports were sorted by CASEID, FDA_DT, and PRIMARYID; for each CASEID, we retained the record with the most recent FDA_DT (and the highest PRIMARYID when ties occurred) (Zou et al., 2024).

### 2.2 Exposure and comparators

The exposure of interest was suzetrigine (including known synonyms/brand identifiers such as VX-548 and JOURNAVX, normalized to the active ingredient for analysis). We focused on reports where suzetrigine was coded as the primary suspect (PS) drug to enhance interpretability and reduce confounding from non-suspect co-medications (Zou et al., 2024).

For comparator-referenced analyses, we selected acetaminophen and ibuprofen as active comparators because they are widely used non-opioid analgesics in similar acute-pain care pathways, providing a clinically relevant background for distinguishing drug-specific reporting patterns.

### 2.3 Outcomes and coding

Adverse events were defined at the MedDRA Preferred Term (PT) level as recorded in FAERS, with optional descriptive aggregation to System Organ Class (SOC) for summarization (Zou et al., 2024). Clinical outcomes were described using FAERS outcome codes (e.g., DE death, HO hospitalization, LT life-threatening, DS disability, OT other serious outcome, RI required intervention), summarized descriptively within the suzetrigine-exposed reports.

### 2.4 Signal detection (disproportionality + bayesian shrinkage)

We used a case/non-case design with 2 × 2 contingency tables for each drug–PT pair, contrasting reporting of a given PT for the target drug versus all other drugs. Disproportionality was quantified using ROR and PRR, and Bayesian approaches using IC (BCPNN) and EBGM-type shrinkage (MGPS/Gamma–Poisson shrinker family) (Zou et al., 2024; Canida and Ihrie, 2017). Signal criteria followed widely used FAERS thresholds to ensure minimum stability and concordance across methods: a ≥3 (minimum case count), ROR lower 95% CI > 1, PRR ≥ 2 with χ^2^ ≥ 4, IC025 > 0, and EBGM05 > 2 (Zou et al., 2024).

To address multiplicity across many PT hypotheses, we implemented false discovery rate (FDR) control (BH procedure) as the primary correction and Bonferroni family-wise error control as a sensitivity anchor (Lu et al., 2023; Huang et al., 2025). Primary statistical significance was defined using BH-FDR–adjusted p values; Bonferroni-adjusted results were reported to evaluate robustness.

### 2.5 Comparator-referenced empirical bayes (EB) profiling

To better characterize drug-specificity within an analgesic context, we implemented a comparator-referenced empirical Bayes (EB) framework informed by the flexible EB approach proposed by Tan et al. (2025).

Briefly, we constructed an analgesic-focused reporting background including suzetrigine, acetaminophen, ibuprofen, and an “other drugs” category. For each PT, the EB model estimates a posterior distribution for the signal strength by borrowing information across the full table (shrinkage), thereby stabilizing estimates for sparse PT counts. We summarized PT-level EB strength using posterior quantiles (e.g., EB q05) based on Monte Carlo posterior draws (3,000 draws in our implementation), and compared the same PT’s EB distributions across exposures. PTs were prioritized when they showed a stronger lower-tail EB signal under suzetrigine than under acetaminophen/ibuprofen and the general background, consistent with a pattern more likely driven by suzetrigine rather than analgesic-class/indication-related reporting.

### 2.6 External validation (triangulation)

To validate and contextualize the prioritized adverse event signals identified from FAERS analyses, we conducted external triangulation by cross-referencing high-priority preferred terms (PTs) with evidence from peer-reviewed literature and global pharmacovigilance data. Literature sources included clinical trials (e.g., Phase II/III randomized controlled trials), meta-analyses, systematic reviews, and case reports, sourced via targeted searches in databases such as PubMed, EMBASE, Web of Science and Google Scholar using keywords related to suzetrigine (e.g., “VX-548,” “JOURNAVX,” “adverse events,” “safety profile”). We extracted details on reported adverse reactions, incidence rates, severity, and mechanistic explanations where available.

Additionally, we incorporated spontaneous reporting data from the World Health Organization (WHO) VigiBase database as of February 2026, focusing on report counts for suzetrigine-associated PTs within relevant system organ classes (SOCs). VigiBase data were queried for absolute report numbers and proportional contributions to total adverse drug reactions (ADRs) for suzetrigine, providing a global perspective to complement U.S.-centric FAERS findings.

This triangulation aimed to assess consistency across sources, prioritize signals with multi-source support and identify gaps for signals lacking corroboration, thereby enhancing the clinical interpretability of detected signals.

### 2.7 Statistical software

All statistical analyses were performed using R statistical software version 4.4.0. The comparator-referenced empirical Bayes (EB) profiling, which enables simultaneous signal detection and signal strength estimation, was implemented according to the flexible empirical Bayesian methodology described by Tan et al. (2025), utilizing the dedicated pvEBayes package (Tan et al., 2025). For each preferred term, 3,000 posterior draws were generated via Markov chain Monte Carlo sampling to obtain empirical Bayes geometric mean (EBGM) estimates and the corresponding lower 5% quantile (EB q05). Custom R functions were developed for comparator referencing (against acetaminophen and ibuprofen), classification of suzetrigine-unique signals, and generation of the comparative EB q05 heatmap.

## 3 Result

### 3.1 Report characteristics

From 2025Q1 to 2025Q3, a total of 405 FAERS reports listed suzetrigine as the primary suspect drug. Reports were predominantly submitted from the United States (100%). Females accounted for 42.5%, males 28.4%, and sex was unknown in 29.1%. Consumers were the most common reporter type (53.1%), followed by physicians (17.0%) and unknown reporter type (28.9%) (Table 1).

**TABLE 1:** Baseline characteristics of adverse event reports associated with suzetrigine in the FDA adverse event reporting system.

Category	Attribute value	Count (n)	Percentage (%)
Country	US	405	100.00
	Other	0	0.00
Gender	Female	172	42.47
	Male	115	28.40
	Unknown	118	29.14
Reporter type	Consumer	215	53.09
	Physician	69	17.04
	Pharmacist	4	0.99
	Unknown	117	28.89
Age group	<50 years	43	10.62
	≥50 years	116	28.64
	Unknown	246	60.74

### 3.2 Reported outcomes

**FIGURE 1:**
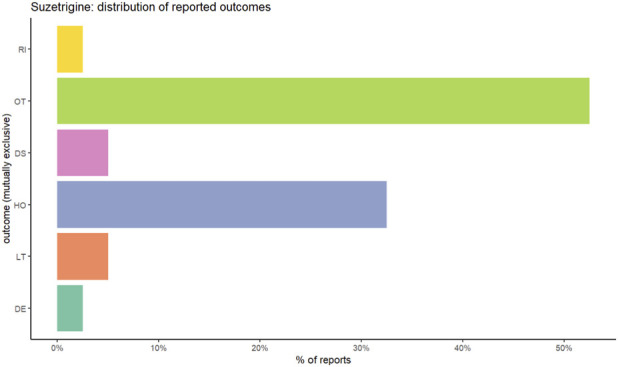
Distribution of reported outcomes for suzetrigine-associated adverse events in FAERS (Outcome codes are case-level designations and cannot be attributed to individual preferred terms within multi-PT cases).

The distribution of reported outcomes was as follows: Other serious (OT) was the most frequent (52.5%), followed by Hospitalization (HO) (32.5%). Less common outcomes included Disability (DS) (5.0%), Life-threatening (LT) (5.0%), Death (DE) (2.5%), and Required intervention to prevent permanent impairment/damage (RI) (2.5%) (Figure 1).

### 3.3 Signal overview and robustness (BH–FDR vs. bonferroni)

At the MedDRA PT level, 19 PTs met the BH–FDR criterion, of which 14 PTs remained significant after the more conservative Bonferroni correction, supporting robustness to multiplicity control. Signals were concentrated in nervous system disorders (7/19) and psychiatric disorders (3/19), followed by skin/subcutaneous tissue disorders (2/19) and musculoskeletal/connective tissue disorders (2/19) (Table 2). The table is sorted by Benjamini–Hochberg FDR-adjusted p-values in ascending order.

**TABLE 2:** Disproportionality signals for suzetrigine in FAERS.

Socs	pt	a	chi_sq	ROR	PRR	IC025	EBGM05	BH–FDR p-value	Bonferroni p-value
Nervous system disorders	PARESTHESIA	28	266.92	12.20	11.43	2.96	8.24	8.36E-35	8.36E-35
Injury, poisoning and procedural complications	OFF LABEL USE	84	109.71	3.35	2.86	1.18	2.34	7.72E-21	1.54E-20
Skin and subcutaneous tissue disorders	PRURITUS	43	96.27	4.27	3.93	1.52	3.01	1.93E-17	5.80E-17
Nervous system disorders	BURNING SENSATION	13	127.56	12.10	11.74	2.75	7.35	6.99E-17	2.80E-16
Musculoskeletal and connective tissue disorders	MUSCLE SPASMS	19	74.45	5.95	5.72	1.85	3.88	1.73E-12	8.65E-12
Nervous system disorders	DIZZINESS	32	56.17	3.63	3.42	1.26	2.53	1.10E-10	6.59E-10
Skin and subcutaneous tissue disorders	SKIN BURNING SENSATION	10	70.74	9.17	8.97	2.25	5.27	1.79E-10	1.25E-09
Nervous system disorders	SOMNOLENCE	18	53.44	4.91	4.73	1.56	3.18	1.38E-09	1.11E-08
Cardiac disorders	PALPITATIONS	12	48.98	6.05	5.90	1.73	3.64	2.40E-08	2.16E-07
Psychiatric disorders	ABNORMAL DREAMS	5	67.23	15.62	15.44	2.67	7.31	2.86E-08	2.86E-07
Nervous system disorders	BRAIN FOG	9	38.94	6.28	6.16	1.67	3.53	1.22E-06	1.34E-05
Gastrointestinal disorders	SWOLLEN TONGUE	5	42.33	10.51	10.39	2.10	4.93	3.57E-06	4.64E-05
Nervous system disorders	HYPOAESTHESIA	12	31.54	4.49	4.39	1.30	2.70	5.55E-06	7.76E-05
General disorders and administration site conditions	FEELING ABNORMAL	12	21.30	3.55	3.47	0.97	2.14	0.000234,061	0.003979035
Vascular disorders	FLUSHING	6	16.63	4.60	4.55	1.02	2.31	0.002816539	0.053514235
Psychiatric disorders	EUPHORIC MOOD	3	63.69	23.53	23.36	2.89	8.90	0.004142025	0.082840492
Psychiatric disorders	NIGHTMARE	4	23.12	7.72	7.66	1.51	3.34	0.019627775	0.521774959
Nervous system disorders	RESTLESS LEGS SYNDROME	3	23.46	9.80	9.74	1.64	3.74	0.030986551	0.99156962
Musculoskeletal and connective tissue disorders	MUSCLE TWITCHING	3	18.80	8.21	8.16	1.38	3.13	0.044823886	1

Abbreviations: PT, preferred term; SOCs, system organ classes; a, number of reports; chi_sq, chi-square statistic; ROR, reporting odds ratio; PRR, proportional reporting ratio; IC025, lower 95% CI, of the information component; EBGM05, lower 5th percentile of the empirical Bayes geometric mean; BH–FDR, Benjamini–Hochberg false discovery rate correction; Bonferroni, family-wise error rate correction.

### 3.4 Comparator-referenced EB profiling (acetaminophen, ibuprofen, other-drug background)

**FIGURE 2:**
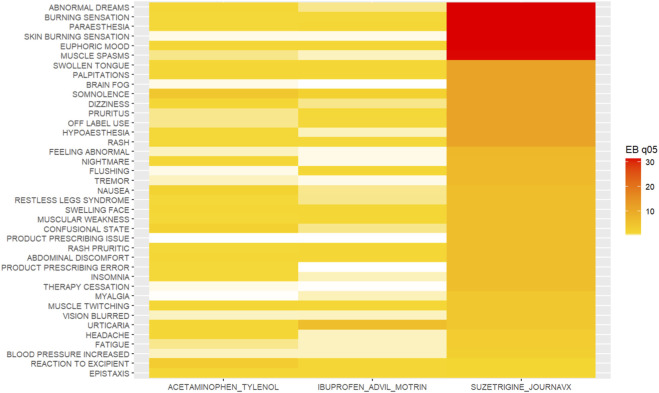
Comparative Heatmap of Empirical Bayes Lower 5% Quantiles (EB q05) for Selected Adverse Event Preferred Terms Across Suzetrigine, acetaminophen, and Ibuprofen.

To characterize drug-specific adverse event signals for suzetrigine relative to comparators, we applied a comparator-referenced empirical Bayes (EB) profiling approach, as described by Tan et al. (2025), which enables simultaneous signal detection and strength estimation in spontaneous reporting data through flexible Bayesian shrinkage. This method incorporated suzetrigine, acetaminophen, ibuprofen, and an “other drugs” background, generating empirical Bayes geometric mean (EBGM) distributions with 3,000 posterior draws for each preferred term (PT) across exposures. Signal thresholds were set at EB q05 < 2 for a suzetrigine‐associated signal, with comparator flags (apap_signal and ibu_signal) indicating whether the same PT met the criterion for acetaminophen or ibuprofen, respectively. For each PT, a higher EB q05 value for suzetrigine reflects a higher co‐reporting frequency of that PT with suzetrigine relative to the two comparators (acetaminophen and ibuprofen) in the spontaneous reporting database. Among all PTs evaluated, the three highest suzetrigine EB q05 values were observed for abnormal dreams, burning sensation, and paresthesia (EB q05 = 31.16 each). The heatmap visualization (Figure 2) depicts EB q05 values, with color intensity scaled from light yellow (low values, indicating minimal signal strength) to dark red (high values, reflecting stronger disproportionate reporting), facilitating visual identification of suzetrigine-enriched PTs relative to comparators. This profiling highlighted neurological and sensory PTs as predominant suzetrigine signals, supporting prioritization for further clinical review (Detailed EB q05 results are provided in the Supplementary Material).

### 3.5 External triangulation

To enhance the clinical credibility of the prioritized signals identified through FAERS disproportionality and empirical Bayes analyses, we triangulated high-priority PTs with external evidence from published literature (including clinical trials, meta-analyses, and case reports) and WHO VigiBase spontaneous reporting data as of February 2026 (Table 3). This cross-referencing focused on the top signals, particularly neurological, sensory, and musculoskeletal events, to assess consistency across sources.

**TABLE 3:** External validation of prioritized suzetrigine adverse event signals using literature evidence and WHO VigiBase data (as of February 2026).

PT	Evidence from literature	Sources	WHO VigiBase data (as of February 2026)
DIZZINESS	Frequently reported in phase II/III RCTs and meta-analyses; one meta-analysis reported reduced risk vs. active controls (RR ≈ 0.57)	1. Burhan et al. (2025); 2. Bertoch et al. (2025)	41 reports in nervous system disorders category, which totals 217 ADRs representing 19% of all reports for suzetrigine
SOMNOLENCE	Based on phase II/III trials, it was classified as a rare adverse reaction that is generally mild to moderate in severity	1. Mach et al. (2025); 2. Pham et al. (2025)	27 reports in nervous system disorders category, which totals 217 ADRs representing 19% of all reports for suzetrigine
PRURITUS	In the phase 2 clinical trials included in the review, suzetrigine-treated patients experienced a slightly higher incidence of pruritus (2.1%) compared with placebo (1.6%) but lower than hydrocodone bitartrate–acetaminophen (3.4%); In this review, pruritus (itching) is described as one of the common adverse events observed with suzetrigine	1. AlDoughaim et al. (2025); 2. Chittoria et al. (2025)	70 reports in skin and subcutaneous tissue disorders category, which totals 164 ADRs representing 14% of all reports for suzetrigine
MUSCLE SPASMS	Common AE: 1.3% in suzetrigine vs. 0.5% placebo in the phase 2 clinical trials	1. AlDoughaim et al., (2025); 2. Chittoria et al. (2025)	35 reports in musculoskeletal and connective tissue disorders category, which totals 68 ADRs representing 6% of all reports for suzetrigine
PARESTHESIA	One post-approval case report noted	Adams et al. (2025)	49 reports in nervous system disorders category, which totals 217 ADRs representing 19% of all reports for suzetrigine
BURNING SENSATION	The review indicates that NaV1.8 is a peripheral sensory neuron channel, and theoretically this may be associated with sensory abnormalities such as numbness, tingling, and burning sensations	Medhat et al. (2026)	20 reports in nervous system disorders category, which totals 217 ADRs representing 19% of all reports for suzetrigine
SKIN BURNING SENSATION	The review indicates that NaV1.8 is a peripheral sensory neuron channel, and theoretically this may be associated with sensory abnormalities such as numbness, tingling, and burning sensations	Medhat et al. (2026)	12 reports in skin and subcutaneous tissue disorders category, which totals 164 ADRs representing 14% of all reports for suzetrigine
HYPOAESTHESIA	The review indicates that NaV1.8 is a peripheral sensory neuron channel, and theoretically this may be associated with sensory abnormalities such as numbness, tingling, and burning sensations	Medhat et al. (2026)	19 reports in nervous system disorders category, which totals 217 ADRs representing 19% of all reports for suzetrigine
EUPHORIC MOOD	No literature support, clinical safety evaluations of suzetrigine did not demonstrate any adverse central nervous system (CNS) or behavioral effects	Osteen et al. (2025)	3 reports of euphoric mood in psychiatric disorders category, which totals 68 ADRs representing 6% of all reports for suzetrigine
ABNORMAL DREAMS	No literature support, clinical safety evaluations of suzetrigine did not demonstrate any adverse central nervous system (CNS) or behavioral effects	Osteen et al. (2025)	7 reports of abnormal dreams in psychiatric disorders category, which totals 68 ADRs representing 6% of all reports for suzetrigine

## 4 Discussion

This comprehensive postmarketing safety analysis of suzetrigine (Journavx, VX-548) reveals a distinctive pattern of adverse events (AEs) that, while largely consistent with its peripheral NaV1.8 mechanism, were not fully captured in the controlled trials. We identified several MedDRA preferred terms (PTs) meeting stringent disproportionality and Bonferroni criteria and corroborated by literature or VigiBase data. Most of these signals cluster into sensory (nervous system), musculoskeletal, and cutaneous events. Importantly, no serious central or addictive effects emerged, consistent with the drug’s peripherally-restricted action. Below, we discuss each cluster in turn to interpret the findings and their clinical implications.

### 4.1 Sensory (neurological) disturbances

A striking finding was the prominent signal for sensory disturbances: paresthesia, hypoaesthesia, burning sensation, and skin burning sensation. These PTs had exceptionally high empirical Bayes lower bounds (EB05) far above comparator analgesics, and constituted a large fraction of reports in the nervous system SOC. In contrast, none of these dysesthesia terms were flagged in the pivotal trials (which instead emphasized pruritus and muscle-related AEs (Rejeev et al., 2025)). This discrepancy likely reflects both the broader real-world population and the drug’s novel mechanism. NaV1.8 channels are expressed almost exclusively in small-diameter dorsal root ganglion (DRG) nociceptors and unmyelinated C-fibers. These channels sustain high-frequency firing during pain transmission; selective blockade by suzetrigine stops nociceptive signals without engaging central Na channels (Pham et al., 2025). However, incomplete or heterogeneous block of peripheral nociceptors can paradoxically produce ectopic or abnormal sensory perceptions (tingling, numbness, burning) as has been well documented with other sodium-channel blockers (e.g., local anesthetics). In other words, dysesthesias and numbness are biologically plausible on-target effects of NaV1.8 inhibition.

Supportive evidence from postmarketing and literature triangulates this conclusion. For example, a recent case report described new-onset paresthesia in a patient started on suzetrigine for postoperative pain, resolving upon discontinuation (Adams et al., 2025). The breadth of sensory AEs is echoed in theoretical reviews: NaV1.8 is a peripheral pain-channel, and its blockade has been linked to “numbness, tingling, and burning” in sensory neurons (Medhat et al., 2026). Notably, WHO VigiBase contains dozens of such reports (e.g., ∼49 reports of paresthesia, 20 of burning sensation, 19 of hypoaesthesia by early 2026), underscoring consistency across global surveillance. In clinical terms, these events have generally been described as mild or transient, but they may be noticeable and distressing to patients. Clinicians should therefore counsel patients that temporary peripheral numbness or burning can occur when pain fibers are blocked, and reassure them that this reflects the drug’s peripheral action rather than a CNS toxicity.

### 4.2 Musculoskeletal effects

Muscle spasms emerged as a robust suzetrigine-specific signal, with EB05 values substantially exceeding those of both comparator analgesics. This finding is directly corroborated by the phase 3 clinical programme: pooled trial data showed muscle spasms in 1.3% of suzetrigine-treated participants versus 0.5% on placebo, characterised as a mild adverse event that did not prompt meaningful rates of treatment withdrawal (AlDoughaim et al., 2025; Bertoch et al., 2025). Global spontaneous reporting data are consistent with this pattern—VigiBase recorded approximately 35 cases of muscle spasm (representing roughly 6% of suzetrigine reports in the musculoskeletal SOC as of February 2026) — and the virtual absence of this signal for acetaminophen and ibuprofen in the comparator-referenced analysis supports a drug-specific rather than analgesic-class attribution.

The mechanistic basis is plausible. NaV1.8 channels are expressed in a subset of proprioceptive and motor-afferent fibres, and tonic inhibition of these channels may perturb sensorimotor reflex arcs or alter calcium handling in muscle tissue, producing benign spasms in susceptible individuals. The occurrence of mild creatine kinase elevation and occasional myalgia in the trial programme further supports a degree of peripheral muscle membrane involvement (Rejeev et al., 2025).

Taken together, the available evidence positions muscle spasms as a reproducible, mechanistically coherent, and clinically manageable on-target effect. That said, FAERS data alone cannot confirm whether spasms encountered in routine clinical practice—follow the same self-limiting course observed in controlled trials. Clinicians should routinely enquire about new-onset muscular symptoms during follow-up, with particular vigilance in patients who have pre-existing muscle disorders or who are receiving concomitant agents with overlapping effects on peripheral excitability.

### 4.3 Cutaneous effects

Skin and subcutaneous events also featured prominently. Pruritus (itch) showed a significant signal and has strong external validation. In the clinical trials, pruritus occurred in ∼2.1% of suzetrigine patients (again higher than placebo but lower than hydrocodone/APAP) (Rejeev et al., 2025). WHO data confirm ∼70 pruritus reports (14% of all ADRs), highlighting it as a consistent drug-associated AE. Mechanistically, NaV1.8 is known to be expressed in cutaneous pruriceptors and C-fibers mediating itch (Pham, et al., 2025). Blockade of these fibers can paradoxically trigger histamine-independent itching, a “class effect” of peripheral sodium-channel modulators. Importantly, suzetrigine’s pruritic effects have been mild and transient in studies; most events resolved without intervention. Rash and urticaria appeared less frequently but were also signal-positive and expected from trial labeling. These cutaneous signals reinforce that suzetrigine’s safety profile is dominated by peripheral sensory and inflammatory pathways, without severe dermatologic toxicity.

### 4.4 Central nervous system–related events

Notably, few central nervous system (CNS) AEs were observed. Dizziness and somnolence did show statistical signals, but with mixed support. Trial data reported less dizziness on suzetrigine (≈4% incidence) than on placebo (6%–7%) (Rejeev et al., 2025). Similarly, somnolence was rare (on the order of <1% and mostly mild) (McCoun, et al., 2025). Nevertheless, FAERS flagged these PTs, and VigiBase had dozens of reports (e.g., 41 dizziness reports in the nervous system SOC, ∼19% of all reports). This discrepancy likely reflects real-world context rather than a direct drug effect. For example, outpatient or non-surgical patients taking suzetrigine may be more prone to report dizziness that could actually be related to pain, hydration status, or concomitant medications. These findings are consistent with suzetrigine’s minimal CNS penetration and lack of opioid-like sedation. As reported in recent reviews, suzetrigine’s adverse events have been “mild and limited to peripheral manifestations”, and “with more than 2,400 participants, no signs of CNS-related impairment, respiratory depression, or addictive behaviors were observed” (Pham et al., 2025). Thus, while dizziness and drowsiness warrant awareness (especially in polypharmacy settings), they do not appear to represent novel neuropsychiatric toxicity.

### 4.5 Euphoric mood and abnormal dreams

EUPHORIC MOOD and ABNORMAL DREAMS warrant separate consideration. EUPHORIC MOOD cleared the BH–FDR threshold but failed to survive Bonferroni correction (p = 0.083), which materially limits confidence in its robustness; ABNORMAL DREAMS, by contrast, survived both correction thresholds, though neither signal has any clinical or preclinical basis in suzetrigine’s pharmacology. NaV1.8 channels are not expressed in central reward circuitry, and the drug exerts no dopaminergic activity; accordingly, the suzetrigine prescribing information and FDA public communications explicitly state that the drug produces no euphoric effect.

The apparent emergence of these signals within a comparator-referenced framework requires methodological explanation rather than simple dismissal. The comparator-referenced EB design is constructed to suppress two sources of spurious elevation: analgesic class-level effects shared across acetaminophen and ibuprofen, and general background reporting noise. What it cannot address is a third source—confounding that arises from the therapeutic effect itself. Pain relief, regardless of the agent achieving it, may generate secondary mood improvement or altered sleep quality in patients who have been suffering from acute pain. This indication-inherent confounding is distributed symmetrically across suzetrigine and both active comparators; it therefore produces no differential signal that the comparator framework can detect and remove. The signals survive the “suzetrigine-unique” filter not because they reflect drug-specific pharmacology, but because the framework operates on between-drug contrasts and has no lever on effects that are uniform across the comparison set. This boundary condition of active-comparator designs in spontaneous reporting has been noted in the methodological literature (Alkabbani and Gamble, 2023), and we now acknowledge it explicitly as a limitation of our approach.

Several additional observations reinforce this interpretation. The absolute case counts are sparse—three VigiBase reports for EUPHORIC MOOD and seven for ABNORMAL DREAMS—and no post-marketing case series, pharmacological mechanistic study, or regulatory communication has raised central mood or sleep effects as a safety concern for this drug class. The intense media coverage surrounding suzetrigine’s approval as the first non-opioid analgesic of its kind may also have heightened patient vigilance toward any subjective change, leading to reports of mood shifts or vivid dreaming that would otherwise go unrecorded—a stimulated-reporting dynamic well documented for newly approved agents (Arora et al., 2017; Hoffman et al., 2014).

Taken together, these observations suggest that EUPHORIC MOOD and ABNORMAL DREAMS are better characterized as signals carrying a high prior probability of indication-inherent confounding than as pharmacologically meaningful findings. The present comparator-referenced design, while effective at removing class-level noise, cannot resolve confounding that operates symmetrically across all analgesic comparators, and the statistical fragility of EUPHORIC MOOD under Bonferroni correction further tempers any causal interpretation. Future surveillance efforts using larger post-marketing datasets—particularly those with richer concomitant medication data—would enable stratified analyses capable of disentangling drug-specific effects from those attributable to pain relief itself, and should be pursued as accumulating real-world evidence matures.

### 4.6 Clinical relevance of signal strength

A high EB q05 value demonstrates disproportionate reporting relative to comparators, but statistical signal strength does not map directly to clinical severity. This distinction is particularly important for highly subjective sensory preferred terms such as paresthesia, burning sensation, and skin burning sensation, which are uniquely susceptible to what may be termed novelty-amplified reporting: patients prescribed a widely publicized first-in-class non-opioid analgesic are arguably more likely to notice, attribute, and report peripheral sensory changes than patients on long-established comparators, independent of any true difference in pharmacological effect magnitude (Hauben and Aronson, 2009; Arora et al., 2017). Elevated EB values for these preferred terms may therefore partly reflect heightened reporting intensity rather than a proportional increase in biological harm.

To place signal strength in clinical context, the identified signals can be stratified into three tiers based on the convergence of mechanistic plausibility, external triangulation, and intrinsic clinical weight. The first tier comprises paresthesia, burning sensation, skin burning sensation, hypoaesthesia, and muscle spasms—signals characterized by strong EB values, direct mechanistic grounding in peripheral NaV1.8 channel biology, consistent corroboration across independent literature and VigiBase, and a clinical nature that warrants active prospective monitoring given their potential to impair function or necessitate treatment modification. The second tier includes pruritus, dizziness, and somnolence—signals supported by significant EB values and external corroboration, but whose intrinsic clinical severity is generally self-limiting, whose pharmacological relationship to NaV1.8 inhibition is less mechanistically specific, and which are therefore more appropriately addressed through patient counseling and routine clinical awareness rather than urgent risk-minimization measures. The third tier comprises euphoric mood and abnormal dreams, which, as discussed above, are statistically fragile, lack external validation, and carry a high prior probability of indication-inherent or stimulated-reporting confounding; these signals are hypothesis-generating only and do not at present warrant clinical intervention.

It should be noted that clinical outcome codes in FAERS—including hospitalization, death, life-threatening events, and disability—are assigned at the case level rather than at the level of individual preferred terms within a report. A single case may encompass multiple adverse events, and the outcome designation reflects the overall clinical course of that case rather than any specific preferred term contributing to it. Attribution of a particular outcome to an individual preferred term is therefore not possible from FAERS data alone. This structural constraint limits fine-grained severity assessment of individual signals and represents a recognized limitation of spontaneous reporting system analyses; prospective registries designed to capture outcomes at the event level would substantially improve the capacity for such stratification in future work.

## 5 Limitations

Several limitations inherent to spontaneous reporting systems and the early post-marketing phase of a novel agent must be acknowledged when interpreting these findings. FAERS and VigiBase data are subject to well-documented reporting biases, including substantial under-reporting of mild or transient events and stimulated reporting (Weber effect) for newly marketed drugs such as suzetrigine. Consequently, true incidence rates cannot be estimated owing to the absence of exposure denominators. Case-level information is frequently incomplete, precluding formal causality assessment (e.g., using Naranjo or WHO-UMC algorithms) and limiting evaluation of de-challenge/re-challenge patterns, dose–response relationships, or confounding by concomitant medications and underlying acute pain etiologies. Although comparator-referenced empirical Bayes profiling and external triangulation with literature and VigiBase data enhanced signal specificity, residual confounding cannot be fully excluded.

All 405 adverse event reports were submitted from the United States, reflecting suzetrigine’s domestic market availability at the time of this analysis; this geographic restriction limits the generalizability of findings to other health systems, ethnic populations, and clinical settings where pain management protocols, prescribing patterns, and spontaneous reporting cultures may differ substantially (U.S. Food and Drug Administration, 2023). Compounding this concern, approximately 29% of reports had unknown sex and nearly 61% had unknown age. The proportion of missing age data is particularly consequential: suzetrigine undergoes hepatic CYP3A4-mediated metabolism, and the safety profile in elderly patients is a clinical priority; subgroup analyses stratified by age or sex are therefore not supportable with the available dataset.

The therapeutic intensity asymmetry between suzetrigine (indicated for moderate-to-severe acute pain) and the active comparators acetaminophen and ibuprofen (primarily used for mild-to-moderate pain) constitutes an additional structural limitation of the comparator-referenced design (Alkabbani and Gamble, 2023). Patients prescribed suzetrigine likely present with higher baseline pain severity and may differ systematically in comorbidity burden, concomitant medication use, and healthcare engagement from those prescribed over-the-counter analgesics, potentially introducing severity-related confounding into disproportionality estimates. This limitation is inherent to the current landscape of non-opioid analgesic development and does not diminish the internal validity of the comparator-referenced approach, but should be borne in mind when interpreting the absolute magnitude of EB values for suzetrigine-unique signals.

At the time of analysis, suzetrigine had been commercially available for only approximately 12 months, restricting detection of rare, delayed-onset, or cumulative adverse events. The subjective nature of several prioritized preferred terms (PTs)—particularly sensory disturbances such as paresthesia, burning sensation, skin burning sensation, and hypoaesthesia—may also be influenced by differential reporting for a first-in-class mechanism of action. Finally, all signals identified remain hypothesis-generating; observational disproportionality analyses cannot establish causality or quantify absolute risk.

## 6 Future work

Future research should address these gaps through targeted, higher-evidence designs focused on the clinical relevance of the identified PTs. Large-scale prospective cohort studies and post-marketing registries are urgently needed to quantify incidence, severity, duration, and risk factors (including age, pain etiology, dose, duration, comorbidities, and potential SCN10 A genetic variants) associated with sensory dysesthesias, muscle spasms, and pruritus (Veluchamy et al., 2025). Dedicated Phase IV randomized controlled trials or enriched safety studies with standardized patient-reported outcome instruments for sensory symptoms, longer follow-up (>14 days), and active comparators (e.g., NSAIDs or multimodal regimens) will be essential to validate or refute these hypothesis-generating signals (Bertoch et al., 2025). Mechanistic investigations employing quantitative sensory testing, neurophysiological recordings, or human experimental models should further clarify how tonic NaV1.8 blockade can produce paradoxical non-histaminergic pruritus or altered sensory percepts via heterogeneous inhibition of pruriceptive C-fibers (Medhat et al., 2026). Studies in special populations—elderly patients, those with hepatic/renal impairment, pediatrics, and individuals transitioning from chronic opioid use—are also required, particularly given suzetrigine’s hepatic metabolism and potential CYP3A4 interactions (McCoun et al., 2025). Finally, integration of real-world evidence from electronic health records, pharmacogenomic profiling, and continued global pharmacovigilance (updated disproportionality analyses at 24- and 36-month milestones) will enable ongoing signal refinement, risk-minimization strategies (e.g., updated labeling or educational tools), and optimization of suzetrigine’s role in opioid-sparing acute pain management (Gatto et al., 2022).

## 7 Conclusion

In conclusion, this comprehensive post-marketing pharmacovigilance analysis, employing FAERS disproportionality metrics, comparator-referenced empirical Bayes profiling, and multi-source external triangulation, reveals a distinct safety signature for suzetrigine dominated by mechanistically plausible peripheral sensory, musculoskeletal, and cutaneous events that largely extend rather than contradict pre-approval trial data. Paresthesia, burning sensation, muscle spasms and pruritus were corroborated by recent literature and WHO VigiBase reports, while psychiatric signals lacking support were appropriately deprioritized. These findings underscore the complementary value of real-world spontaneous reporting for newly approved non-opioid analgesics.

Importantly, all identified signals remain hypothesis-generating in nature. They highlight potential areas for clinical attention and further investigation but do not establish causality or absolute risk. Rigorous confirmation through well-designed prospective cohort studies, long-term safety registries, and adequately powered randomized controlled trials with extended follow-up and detailed adverse event monitoring is essential to validate these observations, quantify their clinical impact, and fully characterize suzetrigine’s benefit–risk profile. As the first selective NaV1.8 inhibitor approved for moderate-to-severe acute pain, suzetrigine offers a promising opioid-sparing alternative. Continued pharmacovigilance combined with targeted clinical research will be critical to ensure its safe and effective integration into multimodal pain management strategies amid the ongoing public health imperative to reduce opioid reliance.

## Data Availability

Publicly available datasets were analyzed in this study. This data can be found here: https://fis.fda.gov/extensions/FPD-QDE-FAERS/FPD-QDE-FAERS.html.
